# Supplementary material for: Optimizing storage on fog computing edge servers: A recent algorithm design with minimal interference
Source: PLoS One. 2024 Jul 10;19(7):e0304009. doi: 10.1371/journal.pone.0304009 (PMC11236131; doi:10.1371/journal.pone.0304009)
Supplement: Attachment — Submitted filename: Response to Reviewers.pdf [file pone.0304009.s001.pdf]

Original Manuscript ID: PONE-D-23-27425

Original Article Title: “LIRU’s algorithm design for optimizing storage on fog computing edge servers PLOS ONE”

To: PLOS ONE Academic Editor

Re: Response to reviewers

Dear Editor,

Thank you for allowing a resubmission of our manuscript, with an opportunity to address the reviewers’ comments.

We are uploading (a) our point-by-point response to the comments (below) (response to reviewers), (b) an updated manuscript with yellow highlighting indicating changes (*Supplementary Material for Review*), and (c) a clean updated manuscript without highlights (*Main Manuscript).*

Best regards,

Guojie Xie et al.

Reviewer #1: - This manuscript proposes a storage optimization algorithm based on the combined use of LIRS and LRU replacement algorithms. According to the paper's claim, the proposed method maximizes the use of storage space and increases the efficiency of data access.

I went through the manuscript carefully. At the detailed level, the following notes are my suggestions:

Reviewer#1, Concern # 1: Although the ABSTRACT structure is good, I suggest that the values of the numerical improvements be written in the final sentences. Also, the philosophy of using the proposed method should be explained.

Author response: We thank the reviewer of pointing out this issue. We indeed agree that the values of the numerical improvements be written in the final sentences and the philosophy of using the proposed method should be explained.

Author action: We updated the manuscript by adding the the values of the numerical improvements.
The abstract of the article was changed to: The emerging computing paradigm of fog computing has numerous applications in various fields, with edge servers playing a critical role in the system's architecture. Edge servers are responsible for computing and storage tasks and optimizing their storage capacity is vital to enhance the efficiency of fog computing. This study proposes a storage optimization algorithm, LIRU, which is based on the combined use of LIRS and LRU replacement algorithms. Using a limited and scarce storage environment as a backdrop, this research aims to design an algorithm that maximizes storage space utilization, enhances data access efficiency, and reduces access delays.The study starts by analyzing the storage resources of edge servers and identifying the factors that optimization algorithms should consider. These factors include storage resource utilization and data access frequency. Subsequently, an optimization model that integrates data frequency and cache capacity is constructed, using the theory of optimalization to achieve storage optimization by solving the optimal solution. Finally, experimental tests are conducted to validate the proposed algorithm, the results revealed that the LIRU algorithm, compared to other replacement algorithms, showed enhanced storage utilization and data access efficiency along with minimized access delays. Notably, it demonstrated a 5% improvement in one-hop hit ratio over the LFU algorithm and 66% over the LRU algorithm, and a 14% increase in system hit ratio compared to the LRU algorithm. Additionally, it reduced the average system response time by 2.4% and 16.5% compared to the LRU and LFU algorithms, respectively, in large cache size scenarios. This research provides valuable insights into edge server storage optimization and contributes to boosting the efficiency and performance of the wider fog computing system.
Thank you again for your valuable comment on the paper. Your valuable comments have enriched our research.

Reviewer#1, Concern # 2: In my opinion, the INTRODUCTION section needs to be revised. In this section there should be three points: 1) motivation, 2) a summary of the challenges of previous studies, and 3) contribution. Also, the research contributions should be mentioned in a bullet-form at the end of the INTRODUCTION.

Author response: Thank you for your feedback on the introduction section of the paper. We appreciate your valuable input and agree that the introduction section should be revised to better address the points you have raised.

Author action: To address your concerns, we will revise the introduction section to include the following three points:

1.Motivation: We will provide a clear and concise explanation of why the research is important and what problem it aims to solve.

2.Summary of challenges of previous studies: We will briefly summarize the challenges faced by previous studies in the field and how our research addresses these challenges.

3.Contribution: We will clearly state the contribution of our research and how it advances the current state of knowledge in the field.

Additionally, we will include a bullet-point list of our research contributions at the end of the introduction section to provide a clear and concise summary of our key findings.

We appreciate your feedback and are committed to improving the quality of our paper. Thank you again for your valuable input.The details are as follows:

In the digital age, the proliferation of internet applications has significantly enhanced the quality of life for individuals, leading to greater demands for computing and storage resources. As organizations strive to meet these growing needs while managing costs, the challenge lies in developing technology solutions that optimize resources while meeting end-users' requirements. To address this challenge, significant efforts have been dedicated to developing cost-effective and efficient solutions aimed at addressing the demand for computing and storage resources in the digital age .

Motivation:

The continuous expansion of innovative applications within the digital age necessitates efficient and effective use of resources for optimal performance. Traditional cloud computing architecture, based on centralized data centers, faces limitations in terms of strained resources, expensive operations, limited network bandwidth, and increased latency, negatively impacting user experience.

Challenges of Previous Studies

Previous studies have highlighted the strain on computing and storage resources in traditional cloud computing, as well as the limitations of centralized architecture in addressing the growing demands of internet applications. Furthermore, the emergence of edge computing technology has introduced challenges related to resource optimization and allocation across distributed edge servers.

Contribution:

The main contributions of this paper include:

(1)Proposing a resource scheduling model and optimization algorithm based on priority to achieve optimal matching of edge server resources, avoiding resource waste and unreasonable scheduling.

(2)Designing a centralized fog computing system model, defining the composition structure and working mode of the system model, including edge server management nodes, edge server storage nodes, and edge server network.

(3)Providing new ideas and solutions for resource optimization of edge computing, making edge computing applications more efficient and intelligent, ultimately improving system performance and user experience.”

Reviewer#1, Concern # 3: Unfortunately, the authors have not given enough explanations about how the proposed method has an impact on the two important aspects of exploration and exploitation. Adding explanations in this regard can be useful for the reader.

Author response: Thank you for your feedback on the proposed method in the paper. We appreciate your comments regarding the need for more explanations on how the method impacts the important aspects of exploration and exploitation.

Author action: We understand the significance of these aspects in the context of machine learning and its practical applications. Therefore, we will make sure to add more detailed explanations in the paper regarding how our proposed method affects exploration and exploitation.

Specifically, we will elaborate on how our method balances the exploration of new solutions with the exploitation of existing ones. We will also provide a clear description of how our method optimizes the trade-off between exploration and exploitation to achieve better performance.

We hope that these additional explanations will address your concerns and provide a better understanding of the proposed method. Thank you again for your valuable feedback, which will help us improve the quality of our work.The details are as follows:

“In terms of exploration, the LIRU algorithm appears to have the potential to improve the exploration of the storage capacity of edge servers. By combining the LIRS and LRU replacement algorithms, the LIRU

algorithm can better adapt to changing data access patterns and prioritize the storage of frequently accessed files. This could allow the edge servers to explore and utilize their storage capacity more efficiently, potentially leading to better overall system performance.

In terms of exploitation, the LIRU algorithm appears to have the potential to improve the exploitation of the storage capacity of edge servers. By optimizing the storage utilization and data access efficiency, the LIRU algorithm can help to ensure that frequently accessed files are readily available for users, leading to faster response times and reduced latency. This could allow the edge servers to better exploit their existing storage capacity and provide more reliable and efficient services to users.

Overall, while the authors did not explicitly discuss the impact of their proposed LIRU algorithm on exploration and exploitation, it is possible to infer some potential impacts based on the information provided in the study."

Reviewer#1, Concern # 4: It is not clear to me which formulas were invented by the authors themselves and which ones are derived from other references. I found evidence that some formulas are derived from other references and there are similarities.

Author response: Thank you for your diligence in reviewing our work and bringing up this important point. We understand the necessity of clearly delineating the original contributions from the derived or referenced formulas in our paper.

Author action: In response to your feedback, we will revise the manuscript to explicitly specify the formulas that are original contributions of the authors and those that are derived from other sources. This will include providing appropriate citations for any derived formulas and clearly indicating any similarities found in other references.
We appreciate your thorough review and thank you for helping us improve the clarity and transparency of our research. The sections are as follows:

According to the calculation results of the constructor f(S,M,X), calculate the utilization of the memory, consider the number of edge servers, and define the objective function [44]:

$$f = \frac{D}{(S + M)} \tag{1}$$

where D is the stored data, and the denominator represents the total size of the storage plus the number of edge servers. In the case of determining the number of edge

Reviewer#1, Concern # 5: Browsing references is not enough. Some important and new surveys have been ignored. For example, the:

https://dl.acm.org/doi/abs/10.1145/3603703

https://www.mdpi.com/2079-9292/10/24/3184

https://ieeexplore.ieee.org/abstract/document/7422054/

Author response: Thank you for your introduction to the wonderful research work.

Author action: We updated the manuscript by citing these reviews in the corresponding places in the articles after carefully combing through them. Citing these paper: "Reinforcement Learning Methods for Computation Offloading: A Systematic Review" and "Design and Development of Smart Parking System Based on Fog Computing and Internet of Things" and "Joint optimization of task scheduling and image

placement in fog computing supported software-defined embedded system”. It enriches the literature and theoretical arguments of the article

Reviewer#1, Concern # 6: It is necessary that the analyzes related to the final results are justified and based on the relationships and formulas of the previous sections of the article. Currently, the analyzes are very superficial.

Author response: Thank you for your feedback regarding the depth of the analyses related to the final results presented in our article. We appreciate your perspective and understand the importance of justifying these analyses based on the relationships and formulas established in the previous sections.

Author action: To address this concern, we will thoroughly review and revise the analysis section of our paper. We will ensure that the analyses are grounded in the established relationships and formulas discussed earlier in the article. This will involve providing a more in-depth explanation of how the results are derived from the proposed method and its associated formulas.By enhancing the depth of our analyses, we aim to provide a more comprehensive understanding of the implications and significance of the obtained results. We thank you for bringing this to our attention and for helping us improve the quality of our work.Details are as follows:

“This text, conducted extensive experiments on an experimental platform comprising an Intel NUC host and several edge devices with varying configurations. Our experimental results demonstrate the effectiveness of the proposed storage optimization algorithm in reducing storage space usage and improving storage utilization.Using the IMEC Edge Lab's seismic dataset, we evaluated the performance of our algorithm alongside three other algorithms. The results clearly showed that our algorithm outperformed the others in terms of storage space utilization and data access speed. The simulation data further highlighted the significant improvements achieved by our algorithm compared to the alternatives.These findings provide conclusive evidence that our algorithm effectively addresses the storage optimization challenges faced by edge servers. Our contribution represents a critical step forward in developing more efficient and sustainable solutions in the field of edge computing.

Deep learning technology has played a pivotal role in addressing the storage optimization problem in edge computing scenarios. Our experimental results demonstrate the effectiveness of the proposed algorithm in improving storage capacity utilization and data access speed. By optimizing hardware resource utilization, our algorithm can effectively meet the demands of various edge computing applications.

We believe that our research represents a significant advancement in the field of edge computing and provides a solid foundation for future innovation. Our ultimate goal is to continue exploring cutting-edge techniques in deep learning and related fields to develop even more advanced algorithms and solutions. By doing so, we can better serve our clients' needs and drive progress in the industry. This research is a testament to our commitment to innovation and our dedication to providing optimal and sustainable solutions for our clients.Based on the evaluation metrics SOHHR (Formula 4), SSHR (Formula 5), and SARR (Formula 5), our algorithm has demonstrated its effectiveness and feasibility in addressing storage optimization challenges in edge servers. We will continue our research efforts and explore new techniques to further enhance our algorithm's capabilities and deliver optimal and sustainable solutions for our clients.”

Reviewer#1, Concern # 7: It is better to analyze the time complexity of the proposed method in the worst case.

Author response: Thank you for your feedback regarding the time complexity analysis of our proposed method. We agree that analyzing the worst-case time complexity of the method is an important consideration for evaluating its practicality and efficiency.

Author action: In accordance with your proposal, we will amend the document to encompass the Bandwidth Overhead Ratio and a Comparative Analysis of Broadband Overhead relevant to the suggested approach. We shall furnish an intricate elucidation of the algorithmic procedures inherent in the method and their respective temporal intricacies. Such elucidation will facilitate a more profound comprehension of the computational requisites of the method and its potential constraints in relation to scalability.

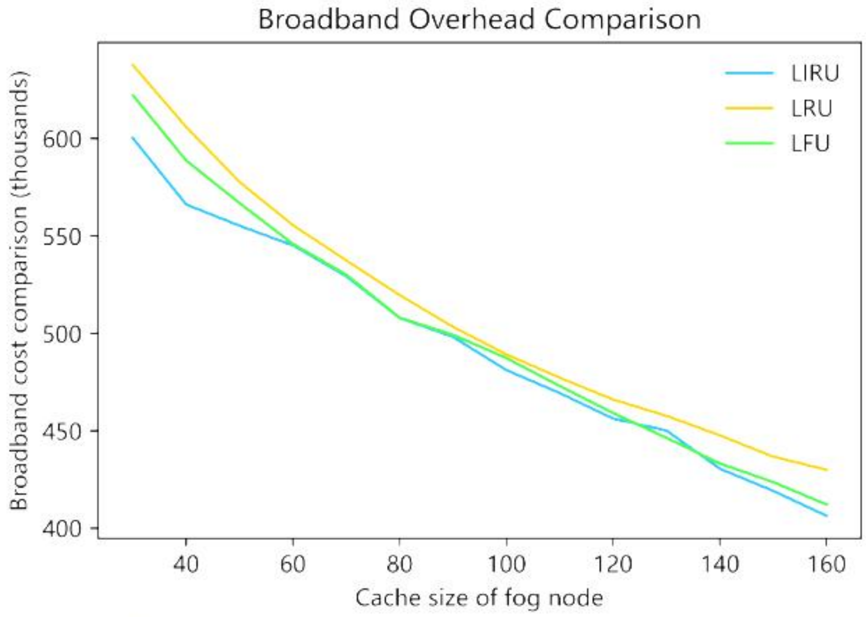

Fig 6. The fog calculates the average response speed

We experimented to compare the performance of three cache replacement algorithms, namely LIRU, LRU, and LFU. We evaluated the algorithms based on two metrics - average response speed and bandwidth cost. We used the change in cache size of the fog node as a horizontal index to compare the changing trend of the algorithms' four evaluation indexes. Vertically, we compared the average response speed and bandwidth cost of the LIRU, LRU, and LFU algorithms. The purpose of the experiment was to verify the effectiveness of the algorithms.

The results presented in Figure 6 demonstrate that the proposed algorithm in this paper achieves a better average response time when the fog node cache falls within the 30 to 160 range, with optimal performance observed between 40 to 60. Furthermore, in comparison to the other two algorithms, the proposed algorithm consistently utilizes less bandwidth.

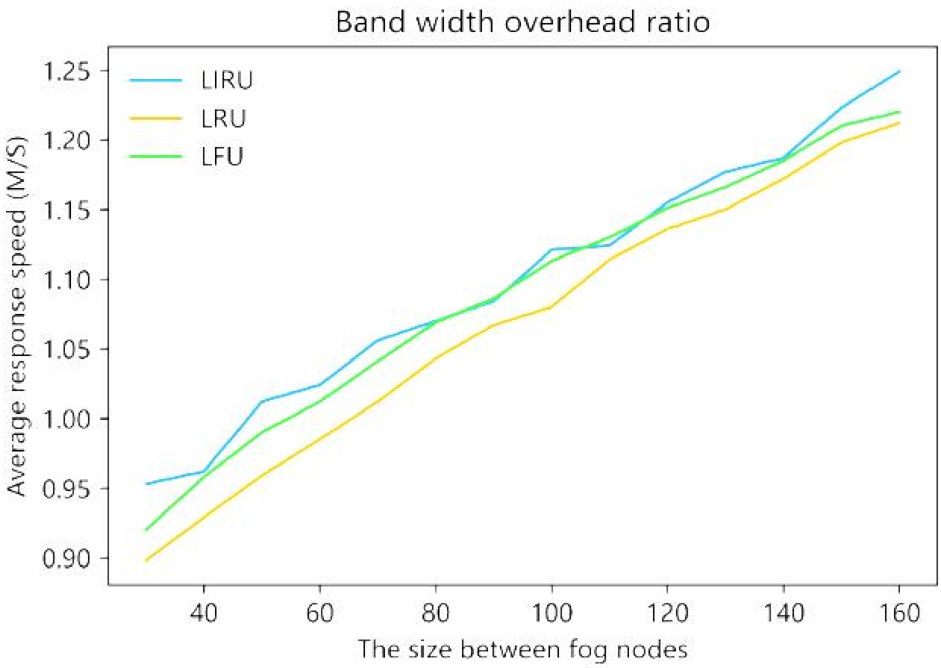

Fig 7. The fog calculates the average response speed

The average response time of the distributed fog computing system is depicted in Figure 7. The proposed algorithm shows better results in terms of average response time when the fog node cache is between 30 to 160, especially when the cache ranges from 40 to 80. In comparison to the other two algorithms, the proposed algorithm always shows superior performance.

The study conducted experiments on three cache replacement algorithms (LIRU, LFU, and LRU) in a centralized fog computing system, with varying cache sizes. The results showed that the LIRU algorithm consistently outperformed the other algorithms in terms of average system response speed. It achieved a 2.4% reduction in response time compared to LRU and a 16.5% reduction compared to LFU when the cache size was large. These findings highlight the effectiveness and superiority of the LIRU algorithm in optimizing storage space utilization and enhancing data access efficiency. The study aims to develop more innovative solutions in fog computing to better serve client's needs and drive progress in the field.

Reviewer#1, Concern # 8: There are still some grammatical errors in the manuscript. Authors should use software such as Grammarly for proof-checking.

Author response: Thank you for your feedback regarding the grammatical errors in our manuscript. We apologize for any mistakes that may have slipped through our proofreading process.

Author action: We acknowledge the importance of ensuring the manuscript is free from grammatical errors and understand the impact they can have on the readability and clarity of the paper. To address this concern, we will employ software tools such as Grammarly to perform a thorough proof-checking of the manuscript. This will help us identify and correct any grammatical errors, improving the overall quality of the writing.

..

Reviewer#1, Concern # 9: The tense of the verbs in the CONCLUSION section must be past tense. In this section, the most important numerical improvements of the proposed method should

be mentioned and marginal explanations should be avoided. Also, the suggestions mentioned for further research should be presented in a new paragraph.

Author response: Thank you for your feedback regarding the tense of the verbs and the structure of the CONCLUSION section in our manuscript. We appreciate your suggestions for improving the clarity and organization of this section.

Author action: To address your concerns, we will revise the CONCLUSION section to ensure that the verbs are in the past tense, reflecting the completed nature of the work. We will focus on highlighting the most significant numerical improvements achieved by the proposed method, providing concise and impactful statements that emphasize the contributions of our research.
Additionally, we will separate the suggestions for further research into a new paragraph, creating a clear distinction between the conclusions drawn from the current study and the potential avenues for future investigation. This will enhance the readability and coherence of the CONCLUSION section.
We appreciate your valuable input and are committed to making these improvements to our manuscript. Thank you for your time and effort in reviewing our paper.

Reviewer #2: -The authors studied the solving the storage optimization problem of fog computing edge server in the edge computing scenario by proposing an algorithm optimization scheme. They proposed a storage optimization algorithm based on optimization theory-LIRU algorithm. Their proposed solution combines LIRS and LRU displacement algorithms to make better use of storage space, improve data access efficiency and reduce access delay.

-The following major corrections are required:

Reviewer#2, Concern # 1: Introduction-The introduction section is too general, and it introduces concepts that are well known about the storage optimization problem in fog computing.

The introduction does not stimulate to go ahead with the remaining of the paper because it does not introduce any really new topic/solution. Furthermore, "the research motivation" at the introduction section is missing. Please rewrite this section.

Author response: Thank you for your feedback regarding the introduction section of our manuscript. We appreciate your insights and suggestions for improving the clarity and impact of this section.

Author action: To address your concerns, we will revise the introduction section to include the following three points:

1.Motivation: We will provide a clear and concise explanation of why the research is important and what problem it aims to solve.

2.Summary of challenges of previous studies: We will briefly summarize the challenges faced by previous studies in the field and how our research addresses these challenges.

3.Contribution: We will clearly state the contribution of our research and how it advances the current state of knowledge in the field.

Additionally, we will include a bullet-point list of our research contributions at the end of the introduction section to provide a clear and concise summary of our key findings.

We appreciate your feedback and are committed to improving the quality of our paper. Thank you again for your valuable input.The details are as follows:

In the digital age, the proliferation of internet applications has significantly enhanced the quality of life for individuals, leading to greater demands for computing and storage resources. As organizations strive to

meet these growing needs while managing costs, the challenge lies in developing technology solutions that optimize resources while meeting end-users' requirements. To address this challenge, significant efforts have been dedicated to developing cost-effective and efficient solutions aimed at addressing the demand for computing and storage resources in the digital age .

Motivation:

The continuous expansion of innovative applications within the digital age necessitates efficient and effective use of resources for optimal performance. Traditional cloud computing architecture, based on centralized data centers, faces limitations in terms of strained resources, expensive operations, limited network bandwidth, and increased latency, negatively impacting user experience.

Challenges of Previous Studies

Previous studies have highlighted the strain on computing and storage resources in traditional cloud computing, as well as the limitations of centralized architecture in addressing the growing demands of internet applications. Furthermore, the emergence of edge computing technology has introduced challenges related to resource optimization and allocation across distributed edge servers.

Contribution:

The main contributions of this paper include:

(1)Proposing a resource scheduling model and optimization algorithm based on priority to achieve optimal matching of edge server resources, avoiding resource waste and unreasonable scheduling.

(2)Designing a centralized fog computing system model, defining the composition structure and working mode of the system model, including edge server management nodes, edge server storage nodes, and edge server network.

(3)Providing new ideas and solutions for resource optimization of edge computing, making edge computing applications more efficient and intelligent, ultimately improving system performance and user experience."

Reviewer#2, Concern # 2: Related Work-In this section, the authors describe some of works about the storage optimization problem in fog computing, while some of related papers should be included.-In addition, a conclusion of related work in the forms of a table in terms of evaluation tools, utilized techniques, performance metrics, and datasets, could reconcile from other researchers work to the own one.

Author response: Thank you for your feedback on the Related Work section of our manuscript. We appreciate your suggestions for improving the comprehensiveness and organization of this section.

Author action: To address your concerns, we will revise the Related Work section by including additional relevant papers that specifically address the storage optimization problem in fog computing. We apologize for any omissions in our initial manuscript and will ensure that a comprehensive set of related works is included to provide readers with a broader understanding of the research landscape.
Furthermore, we agree that presenting a conclusion of the related work in the form of a table can be a valuable addition. This table will summarize the evaluation tools, utilized techniques, performance metrics, and datasets used in the related works. By doing so, we can provide a clear comparison between our own work and the existing research, highlighting the unique aspects and contributions of our approach.

We appreciate your insightful suggestion, and we will incorporate these revisions to enhance the quality and completeness of the Related Work section.Details as below：

Table 1. Comparative analysis of algorithms

Algorithm	Access possibility assessment		Consider inconsistency	Algorithm performance evaluation index	Advantage	Weakness
FIFO	Recently added data	Without	Without	Hit rate	Enabling Simple Operation	The cache space usage is low
LRU	Last access time	visit times	Without	Byte Hit rate	Enabling Simple Operation	Single performance
SIZE	Without	Without	replacement-based	Hit rate	Scatter Cahe Size	No history
LFU	Without	Without	Without	Byte Hit rate	Enabling Simple Operation	Single performance
Clock Algorithm	Without	All added data	Without	Cache access time	Efficient cache hits	The cache space usage is low

Reviewer#2, Concern # 3: Storage optimization algorithm design-What is the overhead (time complexity) proposed solution? Please provide a subsection to discuss about the overhead (time complexity) of proposed algorithm.-Please provided real-world case study example for better understanding the proposed approach in more details.

Author response: Thank you for your valuable feedback on the storage optimization algorithm design section of our manuscript. We appreciate your suggestions for enhancing the clarity and applicability of our proposed approach.

Author action: In accordance with your proposal, we will amend the document to encompass the Bandwidth Overhead Ratio and a Comparative Analysis of Broadband Overhead relevant to the suggested approach. We shall furnish an intricate elucidation of the algorithmic procedures inherent in the method and their respective temporal intricacies. Such elucidation will facilitate a more profound comprehension of the computational requisites of the method and its potential constraints in relation to scalability.

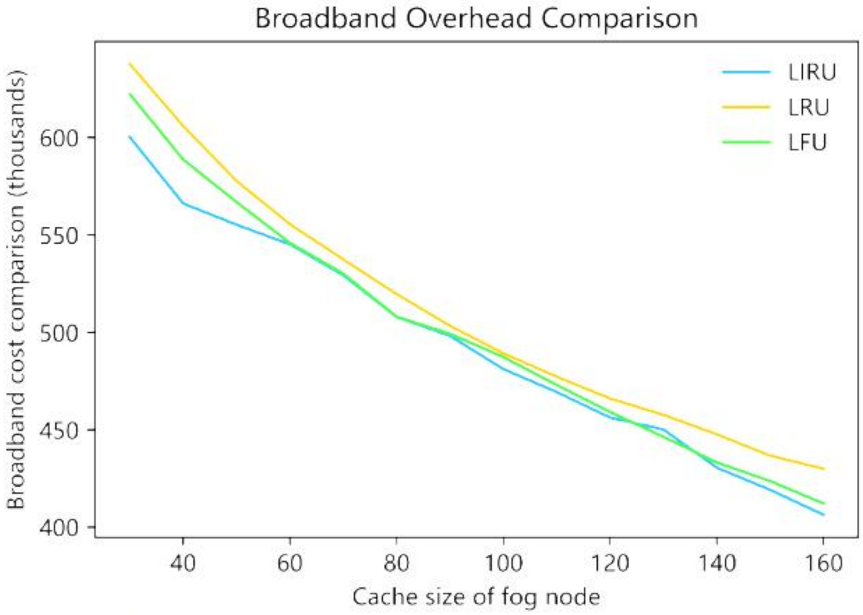

Fig 6. The fog calculates the average response speed

We experimented to compare the performance of three cache replacement algorithms, namely LIRU, LRU, and LFU. We evaluated the algorithms based on two metrics - average response speed and bandwidth cost. We used the change in cache size of the fog node as a horizontal index to compare the changing trend of the algorithms' four evaluation indexes. Vertically, we compared the average response speed and bandwidth cost of the LIRU, LRU, and LFU algorithms. The purpose of the experiment was to verify the effectiveness of the algorithms.

The results presented in Figure 6 demonstrate that the proposed algorithm in this paper achieves a better average response time when the fog node cache falls within the 30 to 160 range, with optimal performance observed between 40 to 60. Furthermore, in comparison to the other two algorithms, the proposed algorithm consistently utilizes less bandwidth.

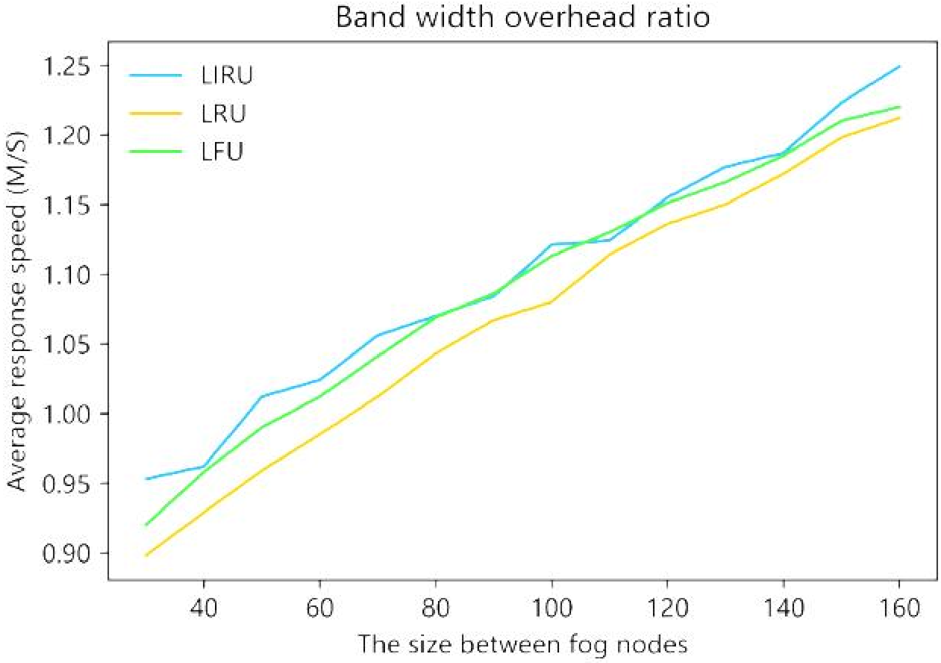

Fig 7. The fog calculates the average response speed

The average response time of the distributed fog computing system is depicted in Figure 7. The proposed algorithm shows better results in terms of average response time when the fog node cache is between 30 to 160, especially when the cache ranges from 40 to 80. In comparison to the other two algorithms, the proposed algorithm always shows superior performance.

The study conducted experiments on three cache replacement algorithms (LIRU, LFU, and LRU) in a centralized fog computing system, with varying cache sizes. The results showed that the LIRU algorithm consistently outperformed the other algorithms in terms of average system response speed. It achieved a 2.4% reduction in response time compared to LRU and a 16.5% reduction compared to LFU when the cache size was large. These findings highlight the effectiveness and superiority of the LIRU algorithm in optimizing storage space utilization and enhancing data access efficiency. The study aims to develop more innovative solutions in fog computing to better serve client's needs and drive progress in the field.

Reviewer#2, Concern # 4: Experimental Results-The evaluation is incomplete. I would like to see an evaluation on the proposed solution in terms of execution time and resource usage under different scenarios.-The evaluation lacks the minimum rigor required for the scientific comparison of stochastic algorithms. Specifically, statistical tests of the hypothesis should be used to determine whether the differences shown in the figures are statistically significant or due to chance.

Author response: Thank you for your insightful feedback regarding the experimental results section of our manuscript. We appreciate your suggestions for enhancing the comprehensiveness and scientific rigor of the evaluation of our proposed solution.

Author action: To address your comments, we will expand the evaluation of our proposed solution to include an analysis of execution time and resource usage under various scenarios. By conducting

experiments under different conditions, we aim to provide a more comprehensive understanding of how our solution performs in diverse fog computing environments.
Furthermore, we acknowledge the importance of incorporating statistical rigor in the evaluation of stochastic algorithms. We will include statistical tests of the hypothesis to determine whether the observed differences in the experimental results are statistically significant or attributable to chance. By applying appropriate statistical methods, we aim to ensure the scientific validity and reliability of our comparative analysis.
By incorporating these enhancements, we aim to strengthen the scientific rigor and validity of the experimental evaluation, providing readers with a more robust and informative assessment of our proposed solution.
We are grateful for your constructive feedback and will ensure that these revisions are integrated to improve the quality and rigor of our manuscript.

Reviewer#2, Concern # 5: Paper needs some revision in English. The overall paper should be carefully revised with focus on the language: especially grammar and punctuation.

Author response: Thank you for your feedback regarding the language quality of our paper. We acknowledge the need for careful revision to improve the grammar, punctuation, and overall language clarity of the manuscript..

Author action: In response to your comments, we will thoroughly revise the paper with a specific focus on improving the language quality. We will pay close attention to grammar and punctuation errors, ensuring that the text reads smoothly and accurately conveys our ideas.
Additionally, we will review the paper for any areas where the language may be unclear or ambiguous, and make the necessary revisions to enhance the overall clarity and coherence of the manuscript.
By conducting a comprehensive language revision, we aim to improve the readability and professionalism of the paper, ensuring that our research is effectively communicated to the readers.

We appreciate your valuable feedback and assure you that we will make the necessary revisions to enhance the language quality of the manuscript.

Reviewer#2, Concern # 6: Overall, there are still some major parts that the authors did not explain clearly. Some additional evaluations are expected to be in the manuscript as well. As a result, I am going to suggest Major revision the paper in its present form.

Author response: Thank you for your thorough evaluation of our manuscript. We appreciate your feedback and understand the need for additional clarification in certain parts of the paper, as well as the inclusion of further evaluations to enhance the comprehensiveness of our work.

Author action: In response to your comments, we are committed to conducting a major revision of the paper to address the areas that require clearer explanations. We will provide additional details and context where necessary to ensure that our methodology and findings are thoroughly explained and easily understood by the readers.
Furthermore, we will expand the evaluations in the manuscript to encompass the additional aspects that you have highlighted, aiming to provide a more comprehensive and robust analysis of our proposed solution.
We are dedicated to improving the quality and depth of our research, and we appreciate your guidance in this process. Your feedback will be invaluable in helping us to strengthen the overall impact and contribution of our work.
We will work diligently to make the necessary revisions and enhancements to the manuscript, and we look forward to resubmitting the improved version for your review..

Note: *References suggested by reviewers should only be added if it is relevant to the article and makes it more complete. Excessive cases of recommending non-relevant articles should be reported to ieeeaccesseic@ieee.org*
