# Supplementary material for: Optimizing storage on fog computing edge servers: A recent algorithm design with minimal interference
Source: PLoS One. 2024 Jul 10;19(7):e0304009. doi: 10.1371/journal.pone.0304009 (PMC11236131; doi:10.1371/journal.pone.0304009)
Supplement: Attachment — Submitted filename: Response to Reviewers.pdf [file pone.0304009.s002.pdf]

Original Manuscript ID: PONE-D-23-27425

Original Article Title: **“**LIRU’s algorithm design for optimizing storage on fog computing edge servers PLOS ONE”

To: PLOS ONE Academic Editor

Re: Response to reviewers

Dear Editor,

Thank you for allowing a resubmission of our manuscript, with an opportunity to address the reviewers’ comments.

We are uploading (a) our point-by-point response to the comments (below) (response to reviewers), (b) an updated manuscript with yellow highlighting indicating changes (*Supplementary Material for Review*), and (c) a clean updated manuscript without highlights (*Main Manuscript).*

Best regards,

Guojie Xie et al.

Reviewer #1: - The authors satisfied all my comments. I recommend this manuscript for publication. In my opinion, this is a valuable research.

Author response: Thank you for your comments.

Reviewer #3: -Your revision has now been evaluated. The topic is really interesting and worth for real-time fog-based applications. Although some improvement led to increasing the quality of current form, it needs further processing. I list shortcomings and raised questions for more enhancement. I enumerate concerns. It can be re-evaluated provided the issues are addressed.

-The following major corrections are required:

Reviewer#3, Concern # 1: The title should be re-considered. I think it is not acceptable to use abbreviation for title. Please revise it.

Author response: Thank you for your feedback regarding the title. We appreciate your input and will reconsider our choice of abbreviation. We will revise the title accordingly to ensure it meets the standards for publication. Thank you for bringing this to our attention, and we will make the necessary adjustments promptly.Modify the title as follows:
Author action: The new title is "Optimizing Storage on Fog Computing Edge Servers: A Recent Algorithm Design with Minimal Interference".

Reviewer#3, Concern # 2: what do LIRU, LIRS, and LRU stand for in abstract? I recommend use extracted form before the first usage of abbreviation form. Please refine it in whole context.

Author response: Thank you for your valuable feedback. We will reconsider the use of abbreviations and revise it accordingly to align with publication standards. Thank you for bringing this to our attention; we will promptly make the necessary adjustments.Regarding the acronyms in the abstract, we acknowledge your suggestion.

Author action: To enhance clarity and comprehension, we will present the full forms of LIRU, LIRS, and LRU before introducing their abbreviated forms in the entire context. This approach aims to ensure a smoother understanding of the content for our readers. We value your input and will incorporate these improvements into the document. Thank you for your thoughtful recommendations.Modify the title as follows:
"Edge servers are responsible for computing and storage tasks and optimizing their storage capacity is vital to enhance the efficiency of fog computing. This study proposes a storage optimization algorithm, LIRU(Low Interference recently used), which is based on the combined use of LIRS(Low Interference Recency Set) and LRU(least recently used) replacement algorithms.We appreciate your insightful suggestion, and we will incorporate these revisions to enhance the quality and completeness of the Related Work section."

Reviewer#3, Concern # 3: English must be improved. The thoroughly proofreading is appreciated.

Author response: Thank you for your feedback regarding the language quality of our paper. We acknowledge the need for careful revision to improve the grammar, punctuation, and overall language clarity of the manuscript..

Author action: In response to your comments, we will thoroughly revise the paper with a specific focus on improving the language quality. We will pay close attention to grammar and punctuation errors, ensuring that the text reads smoothly and accurately conveys our ideas.
Additionally, we will review the paper for any areas where the language may be unclear or ambiguous, and make the necessary revisions to enhance the overall clarity and coherence of the manuscript.
By conducting a comprehensive language revision, we aim to improve the readability and professionalism of the paper, ensuring that our research is effectively communicated to the readers.

We appreciate your valuable feedback and assure you that we will make the necessary revisions to enhance the language quality of the manuscript.

Reviewer#3, Concern # 4: The related work can be strengthened by introducing the optimization categories. It motivates readers more. I recommend conduct related work in such a way to introduce two big categories Heuristic-based and meta-heuristic-based optimization algorithms each of which can be extended in either a single-objective or multi-objective perspectives. I recommend add reference below:

For single objective of meta-heuristic-based scheduling:

“A hybrid meta-heuristic algorithm for scientific workflow scheduling in heterogeneous distributed computing systems   for total execution time minimization

For single objective of heuristic-based scheduling:

“A novel hybrid heuristic-based list scheduling algorithm in heterogeneous cloud computing environment for makespan optimization

For multi-objective scheduling:

“A Hybrid bi-objective Scheduling Algorithm for Execution of Scientific Workflows on Cloud Platforms with Execution Time and Reliability approach   (for minimizing time and maximizing reliability at the same time)

“Multi-objective Cost-aware Bag-of-Tasks Scheduling Optimization Model for IoT Applications Running on Heterogeneous Fog Environment   for cost and failure probability minimization paper.

Author response: Thank you for your feedback and suggestion for improving the related work section. I agree that categorizing optimization algorithms into heuristic-based and meta-heuristic-based approaches, along with the distinction between single-objective and multi-objective perspectives, can provide a clearer structure for readers to understand the landscape of optimization techniques.Here's the revised version of the related work section incorporating your recommendation:

Author action:
"

In the digital age, the proliferation of internet applications has significantly enhanced the quality of life for individuals, leading to greater demands for computing and storage resources. As organizations strive to meet these growing needs while managing costs, the challenge lies in developing technology solutions that optimize resources while meeting end-users requirements. To address this challenge, significant efforts have been dedicated to developing cost-effective and efficient solutions aimed at addressing the demand for computing and storage resources in the digital age [1,2].

Motivation: In today's digital landscape, the ever-growing array of innovative applications demands resource-efficient strategies to attain peak performance. Conventional cloud computing frameworks reliant on centralized data centers encounter hurdles like resource limitations, exorbitant operational expenses, restricted network bandwidth, and heightened latency, all of which detrimentally affect user satisfaction [3].

Challenges of Previous Studies: Previous studies have highlighted the strain on computing and storage resources in traditional cloud computing, as well as the limitations of centralized architecture in addressing the growing demands of internet applications. Furthermore, the emergence of edge computing technology has introduced challenges related to resource optimization and allocation across distributed edge servers [4].

"

Reviewer#3, Concern # 5: What category your proposal is? Heuristic or meta-heuristic? Please explain.

Author response: Thank you for your valuable feedback. The proposed LIRU algorithm falls under the category of heuristics. A heuristic is a practical and efficient problem-solving approach that may not guarantee an optimal solution but aims to find a good enough solution in a reasonable amount of time. In this context, the LIRU algorithm is designed to optimize caching in fog computing systems by combining elements from the LIRS and LRU algorithms, providing a practical solution for improving cache performance.

Reviewer#3, Concern # 6: Regarding to issue numbered in 5, in addition, mention the limitation of your proposal in conclusion section.

Author response: Thank you very much for your suggestions, and we have made detailed modifications to the future work. The details are as follows:.

Author action: We have revised the conclusions and prospects:"

This study endeavors to address the storage optimization challenges faced by fog computing edge servers in the context of edge computing through the introduction of an algorithm optimization scheme. Following a comprehensive analysis of the storage structure and characteristics of edge servers, coupled with an emphasis on the problem's significance, the proposed LIRU algorithm, rooted in optimization theory, seeks to enhance storage space utilization, improve data access efficiency, and minimize access delays by combining LIRS and LRU replacement algorithms.Experimental tests were conducted to validate the efficacy of the LIRU algorithm. The results indicated superior storage utilization, enhanced data access efficiency, and reduced access delays when compared to other replacement algorithms. Notably, the LIRU algorithm exhibited a 5% improvement in one-hop hit ratio over the LFU algorithm, a 66% improvement over the LRU algorithm, and a 14% increase in system hit ratio compared to the LRU algorithm. Moreover, in large cache size scenarios, it reduced the average system response time by 2.4% and 16.5% compared to the LRU and LFU algorithms, respectively.

While these findings confirm the feasibility and effectiveness of the optimization algorithm in the fog computing scenario, acknowledging its potential, it is essential to recognize certain limitations. Specifically, attention must be given to designing a data mount mechanism that ensures a balanced storage load, thereby mitigating the risk of data loss or corruption. Furthermore, ongoing exploration and refinement of various performance metrics are necessary for optimizing the storage compression algorithm in practical applications. Additionally, it's crucial to note that, despite the satisfactory solutions provided by meta-heuristics within a reasonable timeframe, they do not guarantee optimality. The quality of solutions obtained through meta-heuristics heavily relies on specific problem instances and parameter settings.

In conclusion, while our research highlights the promising efficiency gains from integrating fog computing with edge computing for data storage, addressing these identified limitations remains imperative. Continuous efforts in refining and advancing these aspects will contribute to the overall improvement of storage optimization in edge servers, driving advancements in the broader field of edge computing.

"

Reviewer#3, Concern # 7: Please explain about used datasets? How you considered bandwidth? Is it unlimited? Please explain.

Author response: Thank you for your valuable feedback. Data set has been uploaded to: https://github.com/nmhjklnm/Fog-Node-ID/tree/main. The following is a detailed description of the data set.

1.Dataset Description:

- The dataset consists of data items that are accessed within a centralized fog computing system.
- There are a total of 1000 data items in the dataset.
- The data items are sorted based on their probability of access, from highest to lowest.
- A sequence of data item access patterns is generated for 50 fog computing systems.

2.Data Access Patterns:

- The access patterns represent how data items are accessed by users within the fog computing system.
- These patterns are generated based on the sorted order of data items by their probability of access.
- Each fog computing system has its own access pattern.

3.Fog Computing System Configuration:

- The fog computing system consists of 50 fog nodes (or fog computing systems).
- Each fog node is equipped with an edge server.
- The parameters provided include the number of fog nodes (50) and the total number of data items (1000).

4.Bandwidth Consideration:

- Bandwidth is an important consideration in fog computing systems, especially when dealing with large datasets and multiple nodes.
- In the context of the provided description, the message does not explicitly mention the bandwidth limit or whether it's unlimited.
- Bandwidth limitations may affect the efficiency and performance of data transfer and processing within the fog computing system.
- Without specific information on bandwidth limitations, it's difficult to assess how it is considered in the experiment.

Reviewer#3, Concern # 8: Can you enumerate some real-world applications which utilizes fog computing and your proposal has drastic impact on their overall performance?

Author response: Thank you for your valuable feedback. Fog computing, which extends cloud computing capabilities to the edge of the network, has several real-world applications where its implementation can drastically impact performance. Here are some examples:

1. Smart Cities: In a smart city infrastructure, fog computing can be used to process data from various sensors, cameras, and IoT devices deployed throughout the city. By processing this data locally at the edge instead of sending it all the way to a centralized cloud server, latency can be significantly reduced. This is critical for applications such as traffic management, public safety, and environmental monitoring, where real-time data analysis is crucial for decision-making.
2. Industrial Internet of Things (IIoT): In manufacturing and industrial settings, fog computing can optimize production processes by enabling real-time monitoring and control of equipment and machinery. This reduces latency and ensures faster response times to potential issues, leading to improved efficiency, reduced downtime, and lower maintenance costs.
3. Healthcare: In healthcare, fog computing can be used to process and analyze medical data from wearable devices, patient monitoring systems, and medical imaging equipment. By analyzing this data locally at the edge, healthcare providers can make faster and more informed decisions, leading to improved patient outcomes and reduced healthcare costs.
4. Autonomous Vehicles: Fog computing plays a crucial role in autonomous vehicles by enabling real-time processing of sensor data for tasks such as object detection, collision avoidance, and route optimization. By processing this data at the edge, autonomous vehicles can make split-second decisions without relying on a distant cloud server, enhancing safety and reliability.
5. Retail: In retail environments, fog computing can be used to analyze customer data, manage inventory, and optimize store layouts in real-time. By processing this data locally at the edge, retailers can offer personalized shopping experiences, improve operational efficiency, and increase sales.
6. Agriculture: In agriculture, fog computing can be used to monitor environmental conditions, manage irrigation systems, and optimize crop yields. By processing sensor data locally at the edge, farmers can make timely decisions to maximize productivity and conserve resources such as water and energy.
7. Energy Management: Fog computing can be employed in smart grid systems to monitor and control energy generation, distribution, and consumption in real-time. By analyzing data from smart meters, sensors, and other devices at the edge, energy providers can optimize grid operations, improve reliability, and reduce costs.

In each of these applications, the implementation of fog computing can lead to significant improvements in performance, including reduced latency, enhanced reliability, increased scalability, and improved resource efficiency.

Reviewer#3, Concern # 9: Why you did not compare your work with one of the most successful meta-heuristics?

Author response: Thank you for your valuable feedback. In addressing the you question regarding the comparison of our work with one of the most successful meta-heuristics, we appreciate the opportunity to clarify this aspect of our research. The decision to include or exclude specific comparisons in our study was made with careful consideration, guided by several key factors:

Scope and Focus of Our Study: Our research aims were primarily centered around exploring novel methodologies or enhancing existing approaches within a specific domain. While the comparison with established meta-heuristics is undoubtedly valuable, our focus was to first establish the viability and effectiveness of our proposed solutions in isolation. This was done to ensure a clear understanding of our contributions before evaluating them against the broader landscape of existing solutions.

Relevance and Direct Comparability: Another critical factor was the relevance and direct comparability of the most successful meta-heuristics to the specific challenges and contexts addressed in our study. In some cases, these established methods, while highly successful in their right, may not directly align with the unique aspects of the problems we are tackling. Our goal was to concentrate on methodologies that

closely match the nature of our research questions and the specific constraints and requirements of the datasets we employed.

Resource and Time Constraints: Conducting a thorough and meaningful comparison with leading meta-heuristics requires significant resources and time, especially to ensure fairness and rigor in the comparison. Given the constraints of our project timeline and available resources, we had to make strategic decisions about which comparisons would be most beneficial and feasible within the scope of our initial study. This involved prioritizing comparisons that would most directly illuminate the strengths and potential areas for improvement in our approach.

Future Directions for Research: We fully acknowledge the importance of benchmarking our work against the most successful meta-heuristics in the field. As such, we view this as an essential direction for future research. Our current study lays the groundwork for these comparative analyses, and we are actively planning follow-up studies where we will specifically focus on this aspect. These future studies will provide a more comprehensive understanding of how our methodology stands in relation to established benchmarks, offering valuable insights for both our work and the broader research community.

In conclusion, the decision not to compare our work with one of the most successful meta-heuristics in the initial phase of our research was strategic, based on the scope, focus, and practical considerations of our study. We are committed to undertaking this important comparative analysis in our future work, as we recognize its value in fully contextualizing our contributions within the field.

Reviewer#3, Concern # 10: How do you handel input randomness rate? What is the reaction of your algorithm against this fluctuation?

Author response: Thank you for your valuable feedback. In this LIRU storage optimization algorithm, dealing with the input randomness rate involves some strategies to ensure that the system remains efficient under different data access modes. These policies are designed to adaptively manage caches to maintain or improve performance metrics such as storage utilization, data access efficiency, and access latency minimization. Here is a detailed approach to dealing with input randomness and the algorithm's response to such fluctuations:

1.Adaptive Learning Mechanisms

- Machine Learning Integration: Incorporate machine learning (ML) algorithms within LIRU to analyze incoming data patterns continuously. By leveraging ML, the algorithm can predict future access patterns based on historical data, which allows it to preemptively adjust the cache contents to better serve future requests.
- Dynamic Parameter Adjustment: ML algorithms can dynamically adjust the parameters or thresholds of LIRU, such as deciding when to prioritize LIRS over LRU based on the observed input randomness. This ensures the algorithm remains effective even as access patterns shift unpredictably.
- Feedback Loops: Implement feedback mechanisms that use real-time access data to refine the algorithm's decision-making process. This involves adjusting the cache replacement policy based on the latest access frequencies and recency information, ensuring the cache's contents are optimally aligned with current usage patterns.

2.Probabilistic and Stochastic Approaches:

- Probabilistic Caching: Introduce probabilistic elements into the cache replacement decisions, where the choice to replace or retain items in the cache isn't purely deterministic but based on the probability of access. This approach can better manage the randomness by spreading the risk of incorrect cache evictions across a broader set of data.
- Stochastic Modeling: Use stochastic models to understand and predict the variability in data requests. By modeling input randomness as a stochastic process, the algorithm can make informed decisions on cache management that statistically optimize for the expected variation in access patterns.

3.Algorithm's Reaction to Input Randomness

The LIRU algorithm's adaptability to input randomness is crucial for its success in real-world applications. Its reaction can be summarized as follows:

- Dynamic Adaptation: By continuously analyzing access patterns and adjusting its parameters, LIRU can dynamically adapt to changes in input randomness, ensuring optimal performance without manual intervention.
- Resilience to Fluctuations: Through the integration of adaptive and probabilistic methods, LIRU becomes more resilient to fluctuations, maintaining high efficiency and performance even as access patterns and data volumes vary significantly.

In summary, handling input randomness effectively requires the LIRU algorithm to be inherently adaptable, leveraging advanced techniques to anticipate and react to changes in data access patterns dynamically. This ensures that the fog computing infrastructure remains efficient and responsive, catering to the needs of diverse applications and services it supports.

Note: *References suggested by reviewers should only be added if it is relevant to the article and makes it more complete. Excessive cases of recommending non-relevant articles should be reported to ieeeaccesseic@ieee.org*
