# Supplementary material for: Optimizing storage on fog computing edge servers: A recent algorithm design with minimal interference
Source: PLoS One. 2024 Jul 10;19(7):e0304009. doi: 10.1371/journal.pone.0304009 (PMC11236131; doi:10.1371/journal.pone.0304009)
Supplement: Attachment — Submitted filename: Response to Reviewers.pdf [file pone.0304009.s003.pdf]

Reviewer #2:

Section 1: Introduction

-The introduction section is too general, and it introduces concepts that are well known about the optimizing storage on fog computing environment. The introduction does not stimulate to go ahead with the remaining of the paper because it does not introduce any really new topic/solution. Furthermore, "the research motivation…" at the introduction section is missing. Please rewrite this section.

Response: Thank you for your constructive comments regarding the introduction section of our manuscript. We have carefully considered your feedback and agree that the original introduction lacked specificity and a clear research motivation. To address your concerns, we have made the following revisions:

In response to your feedback, we have made the following amendments to the introduction:

1. We have excised superfluous generalities concerning the digital epoch and its associated computational and storage demands, acknowledging these as established knowledge.

2. Fog computing has been introduced as an innovative paradigm to surmount the constraints of conventional cloud computing, emphasizing the distinct optimization challenges it presents.

3. The research motivation has been articulated precisely, underscoring the imperative for enhanced resource scheduling models and optimization techniques within the fog computing milieu.

4. The contributions section has been restructured to more accurately reflect our proposed methodologies' originality and practical significance, thereby providing a coherent foundation for the subsequent sections of the paper. We are confident that these revisions have fortified the introduction by delineating the context more clearly, asserting the novelty of our research, and augmenting the reader's engagement with our findings..

Section 2: Related Work

-In this section, the authors describe some of works about the optimizing storage on fog computing environment, while some of the papers that should have be included are:

https://link.springer.com/article/10.1007/s10586-022-03575-6

https://www.sciencedirect.com/science/article/abs/pii/S2542660523000136

https://link.springer.com/article/10.1007/s10586-021-03283-7

https://onlinelibrary.wiley.com/doi/abs/10.1002/spe.3032

….

-In addition, a conclusion of related work in the forms of a table in terms of evaluation tools, utilized techniques, performance metrics, and datasets, could reconcile from other researchers work to the own one.

Response: Thank you for your valuable feedback on our manuscript. We have carefully considered your suggestions and have updated the Related Work section to include additional references that contribute significantly to the field of storage optimization in fog

computing environments. Specifically, we have added recent studies that are not included in our original manuscript but are relevant to our work. These studies offer various perspectives and methodologies that enrich the discussion on optimizing storage solutions for fog computing.

Moreover, we have included a summary table at the end of the Related Work section. This table provides a concise overview of the evaluation tools, techniques, performance metrics, and datasets employed by the studies we discussed, including the newly added references. This table clearly compares the different approaches and how they relate to our work. We believe these additions will provide a more comprehensive background for readers and help bridge the connection between existing research and our contributions.

We appreciate your guidance in improving our manuscript and hope these revisions meet your expectations.

Section 3: Algorithm Design

-Please provide a sequence diagram to show the interaction steps of the proposed solution according to Algorithm 1.

-What is the overhead (time complexity) proposed solution? Please provide a subsection to discuss about the overhead (time complexity) of proposed Algorithm 1.

-Your proposed solution is very similar to the following papers. What is the difference between proposed model and mentioned papers?

https://ieeexplore.ieee.org/abstract/document/9027842

https://www.igi-global.com/chapter/optimized-storage-and-resource-management-in-fog-computing-paradigm/324589

-Please provided an example (i.e., a real-world case study) for better understanding the proposed approach in more details.

Response: We sincerely thank you for your valuable comments and constructive suggestions. Your feedback has been instrumental in enhancing the quality and clarity of our manuscript. Accordingly, we have implemented the following revisions:

We have incorporated a sequence diagram to facilitate a more intuitive understanding of the LIRU algorithm's operational flow, as depicted in Figure \ref{fig:sequence_diagram}.

A dedicated subsection has been added to delve into the time complexity of the LIRU algorithm. This analysis elucidates the computational overhead and the complexities associated with operations upon cache access.

We have introduced a comparative subsection that juxtaposes our LIRU algorithm with the existing work you referenced. Our approach is distinguished by its dynamic adjustment to access patterns, achieving a nuanced balance between recency and frequency, a dimension not fully explored in the literature reviewed.

To substantiate the practical applicability of the LIRU algorithm, we have presented a real-world case study within the context of fog computing. This study demonstrates the algorithm's efficacy in optimizing cache management and reducing latency for IoT devices. We trust that these revisions have effectively addressed your concerns and significantly bolstered our manuscript's robustness..

Section 4: Experimental results

-The evaluation is incomplete. I would like to see an evaluation on the proposed solution in terms of execution time and cost under different scenarios.

-The evaluation lacks the minimum rigor required for the scientific comparison of stochastic algorithms. Specifically, statistical tests of the hypothesis should be used to determine whether the differences shown in the figures are statistically significant or due to chance.

Response: Thank you for your constructive feedback. In response to your comments, we have made the following revisions to the experimental results section of our manuscript:

1. We have included an evaluation of the proposed LIRU algorithm's execution time and cost under different scenarios. Figures \ref{figure8} and \ref{figure9} have been added to illustrate the algorithm's performance regarding these metrics. These evaluations provide a deeper understanding of the algorithm's efficiency and scalability and its cost-effectiveness in various operational contexts.

2. To ensure the scientific rigor of our comparison, we have performed statistical hypothesis testing on the experimental results. The statistical tests confirm that the differences observed in the performance of the cache replacement algorithms are statistically significant and not due to random variation. The results of these tests are presented in Table \ref{table1}.

We believe these revisions address your concerns and enhance the quality and reliability of our research findings. We appreciate the opportunity to improve our work with your guidance.

Reviewer #3: The revision has underwent significant improvement. In the current form, I recommend for publication. Therefore, I ACCEPT it.

Response: Thank you.
